# Splicing reprogramming of TRAIL/DISC-components sensitizes lung cancer cells to TRAIL-mediated apoptosis

**DOI:** 10.1038/s41419-021-03567-1

**Published:** 2021-03-17

**Authors:** Oliver H. Voss, Daniel Arango, Justin C. Tossey, Miguel A. Villalona Calero, Andrea I. Doseff

**Affiliations:** 1 Department of Molecular Genetics, The Ohio State University, Columbus, OH USA; 2 Department of Medical Oncology and Therapeutics Research, City of Hope National Medical Center, Duarte, CA USA; 3 Department of Physiology and Department of Pharmacology and Toxicology, Michigan State University, East Lansing, MI USA; 4 Present Address: Department of Microbiology and Immunology, University of Maryland School of Medicine, Baltimore, MD USA; 5 Present Address: Laboratory of Receptor Biology and Gene Expression, National Cancer Institute, NIH, Bethesda, MD USA

**Keywords:** Cancer, Molecular biology

## Abstract

Tumor necrosis factor-related apoptosis-inducing ligand (TRAIL) selective killing of cancer cells underlines its anticancer potential. However, poor tolerability and resistance underscores the need to identify cancer-selective TRAIL-sensitizing agents. Apigenin, a dietary flavonoid, sensitizes lung cancer cell lines to TRAIL. It remains unknown, however, whether apigenin sensitizes primary lung cancer cells to TRAIL and its underlying mechanisms. Here we show that apigenin reprograms alternative splicing of key TRAIL/death-inducing-signaling-complex (DISC) components: TRAIL Death Receptor 5 (DR5) and cellular-FLICE-inhibitory-protein (c-FLIP) by interacting with the RNA-binding proteins hnRNPA2 and MSI2, resulting in increased DR5 and decreased c-FLIP_S_ protein levels, enhancing TRAIL-induced apoptosis of primary lung cancer cells. In addition, apigenin directly bound heat shock protein 70 (Hsp70), promoting TRAIL/DISC assembly and triggering apoptosis. Our findings reveal that apigenin directs alternative splicing and inhibits Hsp70 enhancing TRAIL anticancer activity. These findings underscore impactful synergies between diet and cancer treatments opening new avenues for improved cancer treatments.

## Introduction

Lung cancer remains the deadliest of all cancers, and non-small cell lung carcinoma (NSCLC) accounts for 85% of all lung cancer deaths^1,2^. NSCLC is highly resistant to therapy and shows a dismal 5-year survival rate of only 19%, underscoring the need to identify novel anticancer strategies^2–4^.

Tumor necrosis factor-related apoptosis-inducing ligand (TRAIL) is a proapoptotic ligand that has attracted great interest because of its capacity to induce apoptosis selectively of cancer cells^5–7^. TRAIL binds to death receptor 5 (DR5), which recruits Fas-Associated Death Domain (FADD) and inactive caspase-8, forming the DISC complex^8–10^ and leading to caspase-8 activation and apoptosis. Despite the success of TRAIL in preclinical studies, its clinical performance has been rather limited due to the common resistant of primary lung cancer cells to TRAIL monotherapy^6^. Emerging new TRAIL with higher agonistic capacity and reduced systemic toxicity^11^, prompted interest to identify sensitizers to cancer-selective TRAIL therapy contributing to overcome resistance^12^.

TRAIL resistance can be caused by aberrant expression of apoptotic regulators^6^. Decreased DR5 expression and increased expression of DISC inhibitors, cellular FLICE-inhibitory protein (c-FLIP), and heat shock protein 70 (Hsp70), are major drivers of resistance^7,13–15^. Two c-FLIP protein isoforms arise from alternative splicing of *c-FLIP*_*L*_ and *c-FLIP*_*S*_. c-FLIP_S_ protein is commonly upregulated in cancer and interacts with DISC, inhibiting TRAIL-induced apoptosis^13,16^. The chaperone Hsp70 binds to DR5 blocking DISC formation^15^. Silencing of c-FLIP_S_ or Hsp70 sensitizes lung cancer cells to TRAIL-induced apoptosis^13,14^, highlighting the impact of disrupting these molecules in the TRAIL/DISC pathway. Given the specificity of TRAIL in cancer cell killing and the promising results obtained in preclinical models, identification of synthetic or natural compounds that increase TRAIL efficacy has attracted great interest^7^.

Flavonoids are the largest class of plant dietary nutraceuticals^17^, classified into different subclasses, including the flavones. Apigenin (4′,5′,7-trihydroxyflavone), a flavone abundantly found in numerous vegetables, including celery, exhibits anti-inflammatory, and anticarcinogenic activities in lung cancer models^18–23^. In addition, apigenin sensitizes NSCLC and other cancer cells to TRAIL-induced apoptosis^3,24–26^. We previously identified direct targets of apigenin through phage-display library screening coupled with next-generation sequencing (PD-Seq)^27^. Among the high-affinity targets, we identified the RNA-binding proteins Heterogeneous Nuclear Ribonucleoprotein A2/B1 (hnRNPA2) and Musashi 2 (MSI2), which are known key regulators of alternative splicing^28^. We found that apigenin binding to hnRNPA2 reduces its dimerization, resulting in differences in splice variants of hnRNPA2-dependent transcripts in breast cancer cells^27^. Aberrant splicing profiles are common in cancer and have been recognized as important contributors to therapy resistance^29^. However, whether the ability of apigenin to modulate splicing contributes to sensitization to TRAIL remains unknown.

Here, we investigated whether apigenin sensitizes primary tumor-derived lung cancer cells to TRAIL-induced apoptosis and identified the mechanisms responsible for this sensitization. We found that apigenin sensitizes primary lung cancer cells to TRAIL-induced apoptosis by concomitantly reducing the levels of the splice variant c-FLIP_S_ and altering the abundance of DR5 splice variants, resulting in increased DR5 receptor expression, thus contributing to improved TRAIL efficacy. Moreover, association of apigenin with Hsp70 freed the DISC of this inhibitor, increasing the effectiveness of caspase-8 activation. Thus, our findings reveal that dietary apigenin directs splicing and inhibits Hsp70 to promote a two-pronged mechanism—increased death receptor availability and cessation of apoptosis inhibition—that enhances the anticancer efficacy of TRAIL. These findings will have potential impact on considering the use of apigenin or diets rich in apigenin as TRAIL-sensitizing strategies in future clinical studies and uncover molecular targets that warrant further investigation.

## Results

### Apigenin sensitizes primary patient-derived lung cancer cells to TRAIL-induced apoptosis

**Fig. 1:**
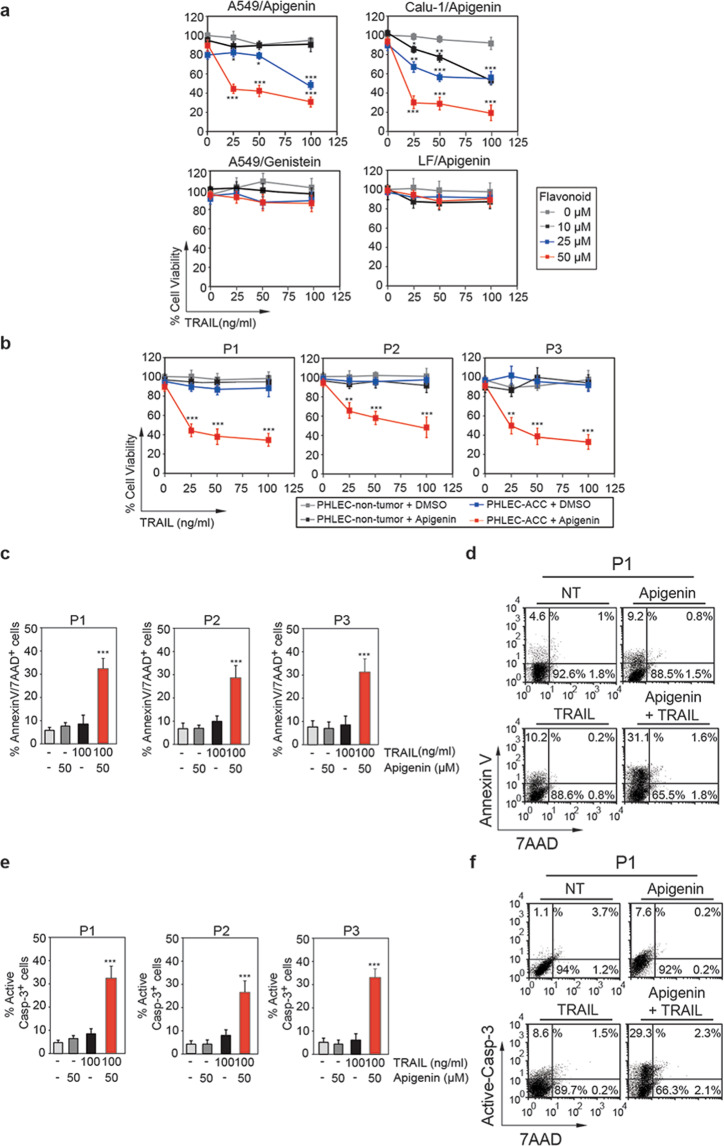
Apigenin sensitizes NSCLC cell lines and primary patient-derived lung cancer cells to TRAIL-induced apoptosis. **a** Cell viability was evaluated using MTT assays in A549 and Calu-1 human NSCLC cell lines and normal nontransformed primary human lung fibroblasts (LF) pretreated with 10, 25, or 50 μM apigenin or DMSO (diluent control, noted as 0) or for 18 h followed by treatment with TRAIL (25, 50, or 100 ng/ml) for additional 6 h. Cell viability was also evaluated in A549 cells pretreated with 10, 25, or 50 μM genistein or diluent for 18 h followed by TRAIL treatment for additional 6 h. **b** Cell viability was evaluated in PHLECs isolated from paired tumor tissue biopsies (PHLEC-ACCs) and nontumor tissue biopsies (nontumor PHLECs) from three subjects (P1–P3) and pretreated with 10, 25, or 50 μM apigenin or diluent for 18 h followed by 25, 50, or 100 ng/ml TRAIL for an additional 6 h. **c**–**f** The percentage of cells stained with AnnexinV/7-AAD (**c**) or active caspase-3 (**e**), which are markers of apoptosis, was evaluated in PHLEC-ACCs isolated from P1 to P3. Cells were pretreated with 50 μM apigenin or DMSO (indicated as -) for 18 h followed by 100 ng/ml TRAIL for 6 h, and then quantification was performed with flow cytometry. A representative flow cytometry plot of cells isolated from P1 stained with AnnexinV/7-AAD (**d**) or active caspase-3 and 7-AAD (**f**). All data are presented as the mean ± SEM (*n* = 3; **P < 0.01, and ***P < 0.001; and analyses were performed with two-way ANOVA).

Previous studies in NSCLC cell lines have administered TRAIL concomitantly with apigenin^25^. Here, we investigated whether apigenin administered prior to TRAIL can sensitize NSCLC cell lines highly resistant to TRAIL. In A549 human lung adenocarcinoma cells (ACCs) and Calu-1 lung epidermoid carcinoma cells, pretreatment with apigenin significantly reduced cell viability at all TRAIL concentrations tested compared to controls (Fig. 1a). Viability was not affected by apigenin alone or the soy-derived isoflavone genistein, another flavonoid with known anticancer activity in NSCLC^30^ (Fig. 1a). Moreover, no effect on the viability of nontransformed primary human lung fibroblasts (LF) was observed (Fig. 1a), suggesting that apigenin specifically affected lung cancer cells. Apigenin and TRAIL treatment-induced apoptosis, as evidenced by staining for Annexin V/7-amino-actinomycin D (7-AAD) and active caspase-3 (Fig. S1).

We next assessed whether apigenin could sensitize patient-derived lung cancer cells to TRAIL therapy. Thus, we used primary human lung epithelial cells (PHLECs) isolated from lung adenocarcinomas (PHLEC-ACCs) and primary human lung epithelial cells isolated from normal adjacent nontumor tissues (nontumor PHLECs) from three independent patients (P1, P2, and P3). Cell isolation resulted in ~90–99% pure lung epithelial cells, evidenced by E-cadherin and β-catenin staining and the almost undetectable staining for the fibroblast marker alpha-smooth muscle actin [αSMA (Fig. S2)]. Apigenin in combination with TRAIL significantly reduced the viability of PHLEC-ACCs in all three patient samples examined at all TRAIL concentrations tested (Fig. 1b). Importantly, apigenin/TRAIL treatment had no effect on the viability of matched nontumor PHLECs (Fig. 1b). Apigenin/TRAIL treatment increased apoptosis, as shown by the threefold increase in cells stained positive for AnnexinV-7-AAD or active caspase-3 compared to cells treated only with apigenin or TRAIL (Fig. 1c–f).

Overall, these results demonstrate that pretreatment with apigenin sensitizes primary patient-derived lung cancer cells and established NSCLC cell lines to TRAIL-induced caspase-3-dependent apoptosis without affecting the viability of nontransformed cells or primary patient-derived nontumor lung epithelial cells.

### Apigenin regulates the expression of specific TRAIL/DISC components in lung cancer cells

**Fig. 2:**
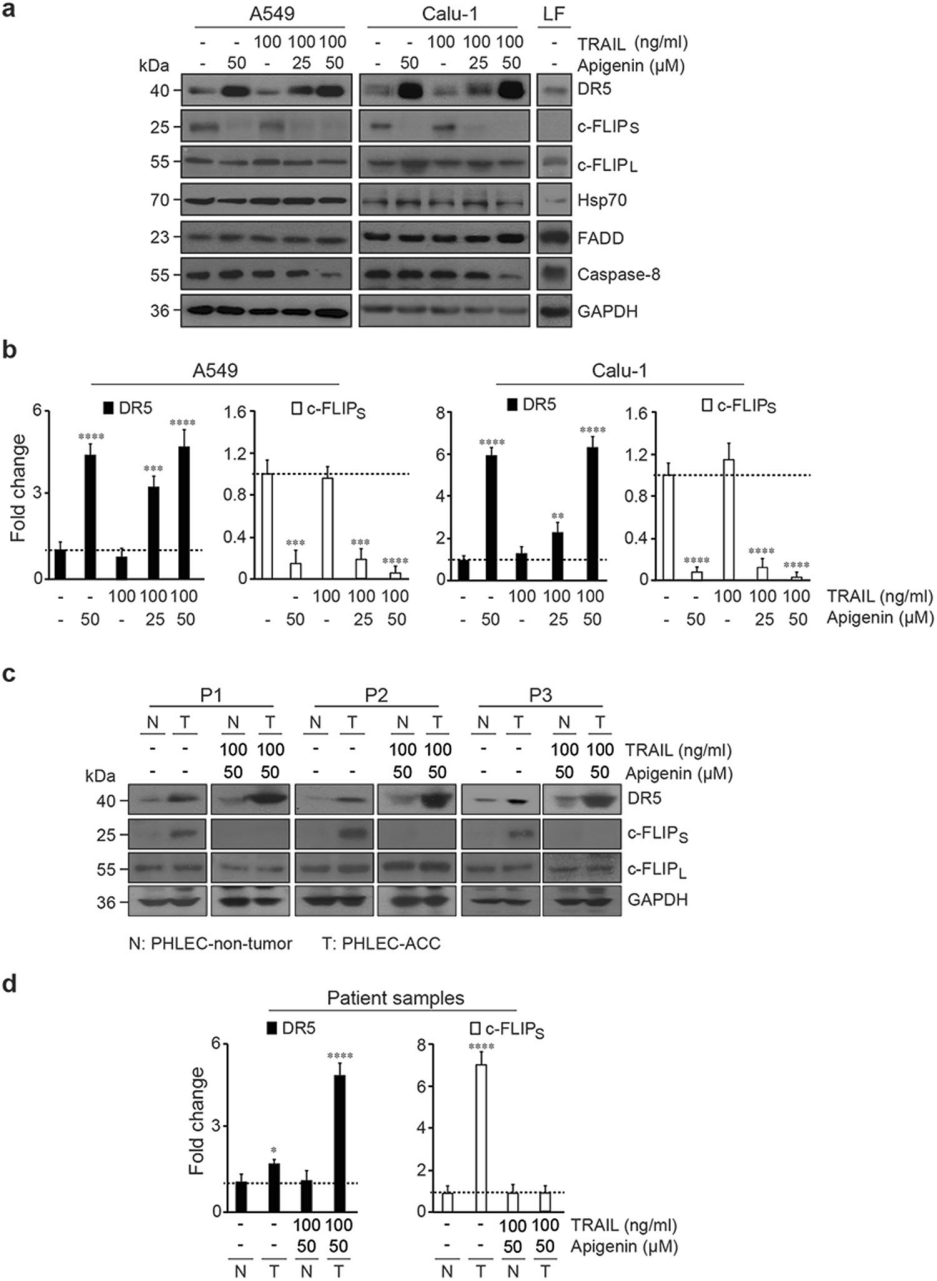
Apigenin increases DR5 and reduces c-FLIP_S_ protein expression in lung cancer cells. Protein levels of DISC components were assessed by western blot (**a**) for A549 and Calu-1 human NSCLC cell lines and normal nontransformed primary human lung fibroblasts (LF) or (**c**) lung epithelial cells isolated from three paired matched normal (N) human biopsies (nontumor PHLECs) and human tumor (T) biopsies (PHLEC-ACCs) (P1–P3). The lung cell lines were pretreated with 25 or 50 μM apigenin for 18 h followed by treatment with 100 ng/ml TRAIL for 6 h, or treated for the whole time with DMSO (-), or 50 μM apigenin alone, or pretreated with DMSO (-) for 18 h followed by TRAIL for 6 h. The primary epithelial cells were pretreated with 50 μM apigenin for 18 h followed by treatment with 100 ng/ml TRAIL for 6 h or with only DMSO (-) for the whole time. Protein lysates were assessed by immunoblot with specific anti-DR5, anti-c-FLIP_S_, anti-c-FLIP_L_, anti-Hsp70, anti-FADD, and anti-caspase-8 antibodies. The same membranes were reblotted with anti-GAPDH antibodies, which functioned as a loading control. The western blots are representative of three independent experiments. **b**, **d** Because DR5 and c-FLIP_S_ appeared to have the most dramatic changes resulting from apigenin treatment, their levels were quantified by densitometry using ImageJ, and the data are presented as the DR5/GAPDH and c-FLIP_S_/GAPDH fold-change ratios. The value for the control treatment (-) was established as 1 to enable easy comparison of relative values. All data are presented as the mean ± SEM (*n* = 3; *P < 0.05, **P < 0.01, ***P < 0.001, and ****P < 0.005; and analyses were performed by two-way ANOVA).

To identify the mechanisms by which apigenin pretreatment sensitizes lung cancer cells to TRAIL-induced apoptosis, we examined the effect of apigenin on the accumulation of key proteins in the TRAIL/DISC pathway. Of the DISC proteins examined, we found that apigenin pretreatment followed by TRAIL altered only DR5, c-FLIP_S_, and caspase-8, while c-FLIP_L_, FADD, and Hsp70 remained at levels comparable to those found in cells treated with DMSO in both A549 and Calu-1 cells (Fig. 2). Apigenin significantly increased DR5 protein levels by ~5-fold both in A549 and Calu-1 cells, independently of TRAIL treatment, compared to controls or nontransformed human primary LF (Fig. 2a, b). These higher DR5 protein levels induced by apigenin, resulted in increased DR5 cell surface availability (Fig. S3a–c). Contrary, no changes on DR4 expression nor cell surface availability were observed (Fig. S3d–g). Similarly, apigenin pretreatment of the highly metastatic advanced lung carcinoma cell line H1299 increased DR5 expression ~5-fold independently of TRAIL treatment (Fig. S4a, S4b) and significantly increased the efficacy of TRAIL in reducing viability (Fig. S4c).

Apigenin decreased c-FLIP_S_ expression fivefold to tenfold in A549, Calu-1, and H1299 cells, independently of TRAIL treatment (Figs. 2a, b and S4a, b). Full-length caspase-8 decreased only in cells treated with both apigenin and TRAIL, possibly because caspase-8 was cleaved in cells undergoing apoptosis (Figs. 2a and S4).

Next, we investigated whether apigenin affected DR5 and c-FLIP_S_ protein levels in patient-derived PHLEC-ACCs. DR5 expression was ~2-fold higher in PHLEC-ACCs than in matched nontumor PHLECs (Fig. 2c, d). A significant ~6-fold increase in DR5 levels was observed in PHLEC-ACCs pretreated with apigenin but not in nontumor PHLECs treated similarly (Fig. 2c, d). As expected, c-FLIP_S_ expression was detectable in PHLEC-ACCs but not in nontumor PHLECs. Apigenin/TRAIL treatment reduced c-FLIP_S_ expression to undetectable levels in PHLEC-ACCs (Fig. 2c, d). By contrast, c-FLIP_L_ levels were similar in PHLEC-ACCs and nontumor cells, and remained unaltered with apigenin and TRAIL (Fig. 2c, d), suggesting that apigenin affects specifically c-FLIP_S_ protein isoform accumulation.

Overall these results suggest that apigenin regulates the expression of DR5 and c-FLIP_S_, key molecules in the TRAIL/DISC pathway, in both established and human primary patient-derived lung cancer cells.

### Apigenin increases DR5 protein levels by shifting *DR5* splice isoform distribution

**Fig. 3:**
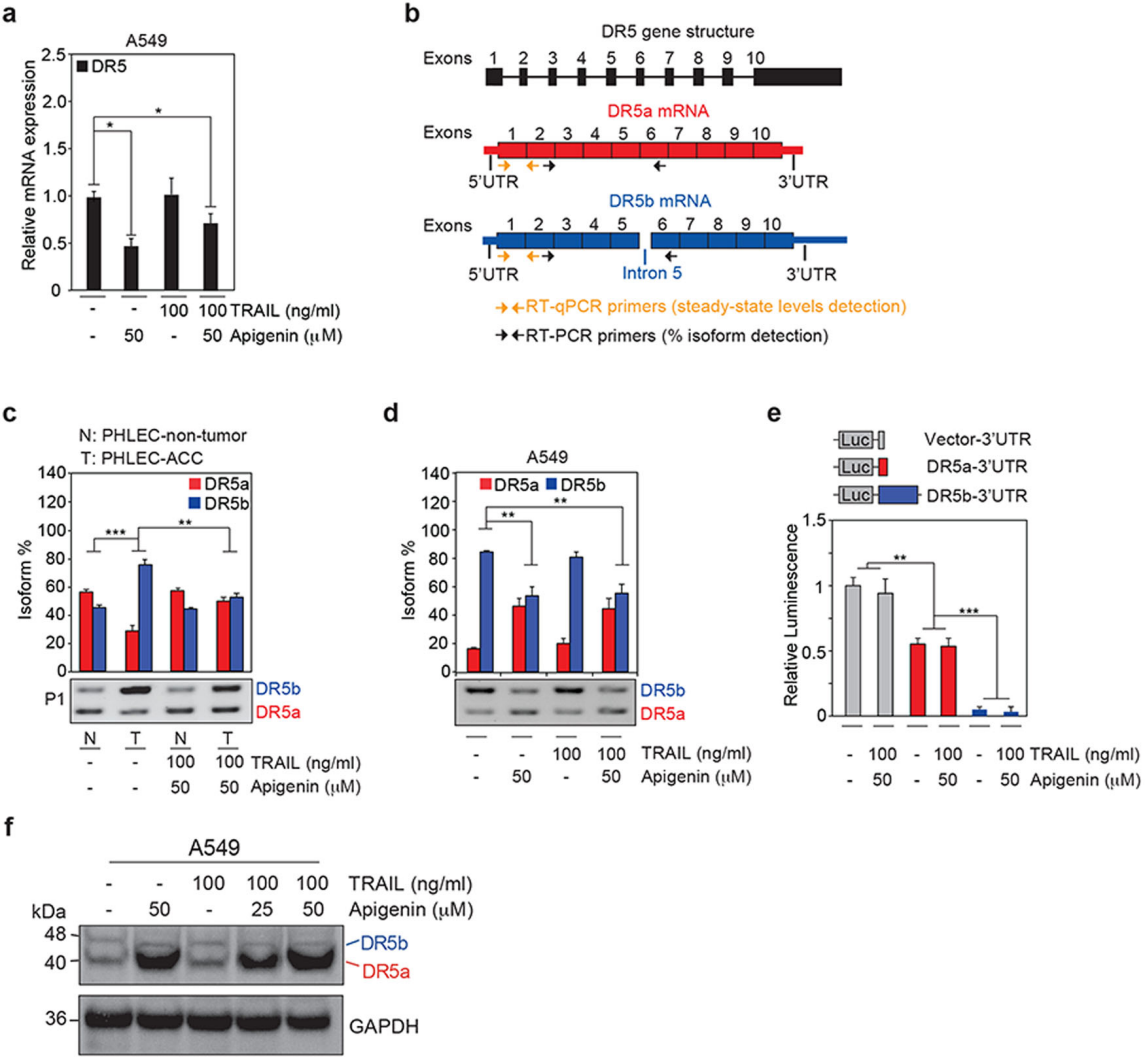
Apigenin modulates alternative splicing of *DR5* and c-FLIP isoforms. **a** A549 NSCLC cells were pretreated with 50 μM apigenin or DMSO (-) for 18 h followed by treatment with 100 ng/ml TRAIL for 6 h; then, the relative steady-state mRNA expression of *DR5* was assessed by RT-qPCR using primers indicated by the orange arrows in (**b**). **b** Schematics of the *DR5* gene structure including introns represented by a line and exons represented by filled numbered boxes. There are two annotated protein-coding *DR5* transcripts, *DR5a* (ENST00000347739.3) and *DR5b* (ENST00000276431.9). The splice isoforms of *DR5* mRNA, *DR5a* (in red), and *DR5b* (blue) with UTRs represented by thinner filled boxes. These two isoforms were amplified by RT-PCR using the same set of primers, indicated by the black arrows, and separated using agarose gel electrophoresis. The amplicon of *DR5b*, which contains intron 5 (lighter blue), is 84 bp larger than the amplicon of *DR5a*. **c** PHLECs isolated from paired nontumor (N) and adenocarcinoma tumor (T) biopsies from human patients (P1–P3) were pretreated for 18 h with 50 μM apigenin or DMSO (-) followed by treatment with 100 ng/ml TRAIL for 6 h; controls were treated only with DMSO (-); then, the alternative splicing of *DR5a* and *DR5b* was determined by RT-PCR using isoform-specific primers (indicated by the black arrows in [**b**]). Quantification of the isoforms observed in the gels was done by densitometry. The percent of each isoform was calculated as follows: (density of one isoform)/(density of the sum of the two isoforms) × 100. Bar plots summarize the quantitation and represent the mean ± SEM (*n* = 3). The picture in the bottom is a representative image of an agarose gel depicting the *DR5a* and *DR5b* isoforms obtained by RT-PCR and separated by gel electrophoresis. **d** The percentages of *DR5b* and *DR5a* isoforms in the same samples used in (**a**) were determined by RT-PCR using isoform-specific primers (indicated by the black arrows in [**b**]). Quantification of the isoforms observed in the gels was done by densitometry as indicated in (**c**). The picture in the bottom is a representative image of an agarose gel depicting the *DR5a* and *DR5b* isoforms obtained by RT-PCR and separated by gel electrophoresis. Bar plots summarize the quantitation and represent the mean ± SEM (*n* = 4). **e** A549 cells transiently transfected with luciferase-tagged *DR5a*-3′UTR, *DR5b*-3′UTR, or luciferase control vector were pretreated for 18 h with apigenin or DMSO and then with 100 ng/ml TRAIL for 6 h, and the relative luminescence activity was measured. Cotransfection with the pCMV-β-Gal plasmid was used for normalization by the corresponding β-Gal activity. **f** Protein levels of the DR5a (40 kDa) and DR5b (48 kDa) isoforms were assessed by western blot in A549 cells using an anti-DR5 antibody from ProScience. The picture is a representative of three independent experiments. Data are expressed as the mean ± SEM (Fig. 3a and d, *n* = 4; Fig. 3c and e, *n* = 3; *P < 0.05, **P < 0.01, and ***P < 0.001; and analyses were performed by two-way ANOVA followed by Tukey’s post hoc analysis).

To study how apigenin increases DR5 protein, we first evaluated the steady-state mRNA levels of *DR5*. Unexpectedly, we found lower *DR5* mRNA levels in A549 cells treated with apigenin alone or in combination with TRAIL than in cells treated with vehicle or TRAIL alone (Fig. 3a).

There are two annotated protein-coding *DR5* transcripts, *DR5a* and *DR5b*. *DR5b* isoform retains intron 5 and has a longer 3′UTR than *DR5a* (Fig. 3b). The function of the retained intron 5 in *DR5b* remains unclear, but differences in *DR5* UTR lengths have been shown to impact the efficiency of DR5 translation in melanoma^31^. Therefore, we speculated that the relative levels of *DR5a* and *DR5b* transcripts would result in differences in translation rates and hence DR5 protein levels. Thus, we evaluated the expression of *DR5a* and *DR5b* splice isoforms. PHLEC-ACCs exhibited significantly higher levels of *DR5b* splice isoform and lower *DR5a* isoform than matched nontumor PHLECs (Fig. 3c). Notably, compared to untreated PHLEC-ACCs, apigenin/TRAIL treatment reduced *DR5b* and increased *DR5a* isoform levels, resulting in a *DR5b/DR5a* isoform ratio resembling that of nontumor PHLECs (Fig. 3c). Similarly, apigenin treatment decreased *DR5b* levels significantly and concomitantly increased *DR5a* transcript expression in A549 cells compared to controls, independently of TRAIL (Fig. 3d). Together, these results demonstrate that primary tumor and nontumor epithelial lung cells have distinct *DR5* splice isoform expression patterns and that apigenin shifts the distribution of *DR5* splice isoforms in tumor cells to a ratio that is similar to that in nontumor cells.

We had shown that, in breast cancer cells, apigenin interacts with the RNA-binding proteins MSI2 and hnRNPA2^27^, which regulate alternative splicing of *DR5* and *c-FLIP*, respectively, and also with Hsp70. To assess if similar interactions occurred in lung cancer cells, we conducted pulldown assays using beads covalently linked to apigenin and A549 cell lysates. We found that MSI2, hnRNPA2, and Hsp70 associated specifically with apigenin beads but not with control beads (Fig. S5), suggesting that apigenin might affect alternative splicing by interacting with these RNA-binding proteins. As expected, the DR5 protein, not identified in our high-throughput screening, was not found associated with apigenin beads, indicating the specificity of the PDSeq approach to identify direct targets of apigenin^27^ (Fig. S5).

We showed that apigenin treatment increased the levels of the *DR5a* transcript, with a shorter 3′UTR (~0.3 kb), and decreased the levels of the *DR5b*, which contains a longer 3′UTR (~2.5 kb). Thus, we next investigated the contribution of the 3′UTR of *DR5* in translation using a luciferase reporter system. We found that the longer *DR5b* 3′UTR almost completely inhibited translation, while the *DR5a* 3′UTR only reduced luciferase translation by 50% of the control, independently of the treatment (Fig. 3e), suggesting that translation efficiency is affected by the length of the 3′UTR, but not by apigenin. Consistently with the increased levels of *DR5a* transcript induced by apigenin, we found higher levels of the DR5a protein isoform in A549 cells treated with apigenin, independently of TRAIL (Fig. 3f).

Together, our findings indicate that apigenin shifts the distribution of splice isoforms; reduces the less efficiently translated (*DR5b*), while increases the levels of the translationally active isoform (*DR5a*), thereby increasing DR5 protein levels and availability.

### Apigenin shifts FLIP cancer-associated splicing profiles to those found in nontumor cells

**Fig. 4:**
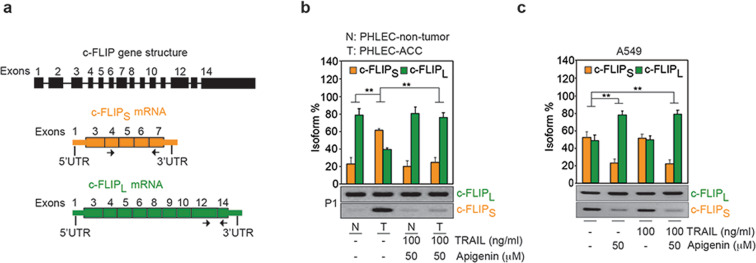
Apigenin modulates alternative splicing of *c-FLIP* isoforms. **a** Schematics of the *c-FLIP* gene structure including introns represented by a line and exons represented by filled numbered boxes. Splice isoforms of *c-FLIP* mRNA, *c-FLIP*_*S*_ (in orange), and *c-FLIP*_*L*_ (in green) including exons and UTRs represented by thinner filled boxes. **b**, **c** The alternative splicing of *c-FLIP*_*S*_ and *c-FLIP*_*L*_ was determined by RT-PCR using isoform-specific primers (indicated by the black arrows in [**a**]). Quantification of the isoforms observed in the gels was done by densitometry. The percent of each isoform was calculated as follows: (density of one isoform)/(density of the sum of the two isoforms) × 100. Bar plots summarize the quantitation and represent the mean ± SEM. The picture in the bottom is a representative image of an agarose gel depicting the *c-FLIP*_*S*_ and *c-FLIP*_*L*_ isoforms obtained by RT-PCR and separated by gel electrophoresis. **b** PHLECs isolated from paired nontumor (N) and adenocarcinoma tumor (T) biopsies from human patients (P1–P3) pretreated for 18 h with 50 μM apigenin or DMSO (-) followed by treatment with 100 ng/ml TRAIL for 6 h or controls treated only with DMSO (-) then, the alternative splicing of *c-FLIP*_*S*_ and *c-FLIP*_*L*_ was determined by RT-PCR using isoform-specific primers. **c** A549 NSCLC cells pretreated with 50 μM apigenin or DMSO (-) for 18 h followed by treatment with 100 ng/ml TRAIL for 6 h, apigenin or TRAIL alone; then, the alternative splicing of *c-FLIP*_*S*_ and *c-FLIP*_*L*_ was determined by RT-PCR using isoform-specific primers (black arrows shown in **a**). All data are expressed as the mean ± SEM. (Fig. 4b, *n* = 3; Fig. 4c, *n* = 4; **P < 0.01; and analyses were performed by two-way ANOVA followed by Tukey’s post hoc analysis).

Next, we studied the mechanisms by which apigenin regulates c-FLIPs protein levels. The shorter c-FLIP isoform (*c-FLIPs*) has an alternate 3′ exon (exon 7), instead of exons 8-14 found in *c-FLIP*_*L*_ (Fig. 4a). *c-FLIPs* inhibits TRAIL/DISC-induced apoptosis^13^, while *c-FLIP*_*L*_ can activate caspase-8-induced apoptosis^32^. Apigenin associates with hnRNPA2 in lung cancer cells (Fig. S4), and this protein regulates *c-FLIP* alternative splicing^33^. Thus, we measured *c-FLIPs* and *c-FLIP*_*L*_ isoforms in patient-derived lung cells using isoform-specific primers (Fig. 4a). Similar to what was observed at the protein level (Fig. 2), *c-FLIPs* transcript levels were significantly higher in PHLEC-ACCs than in matched nontumor PHLECs (Fig. 4b). Apigenin reduced *c-FLIPs* levels in PHLEC-ACCs resembling that of nontumor PHLECs (Fig. 4b). By contrast, *c-FLIP*_*L*_ levels were similar in PHLEC-ACCs and nontumor cells, and they remained unaltered following treatment with apigenin and TRAIL (Fig. 4b). Similarly, apigenin significantly decreased *c-FLIPs* in A549 cells compared to untreated cells, independently of TRAIL (Fig. 4c).

Together, these results demonstrate that primary tumor and nontumor epithelial lung cells have distinct *c-FLIPs* splice isoform expression patterns and that apigenin reduces the levels of *c-FLIPs* splice isoform in tumor cells, thereby shifting the distribution of the splice isoforms to a ratio that is similar to that in nontumor cells.

### Apigenin binding to Hsp70 promotes association of TRAIL/DISC factors, contributing to activation of the apoptotic program

**Fig. 5:**
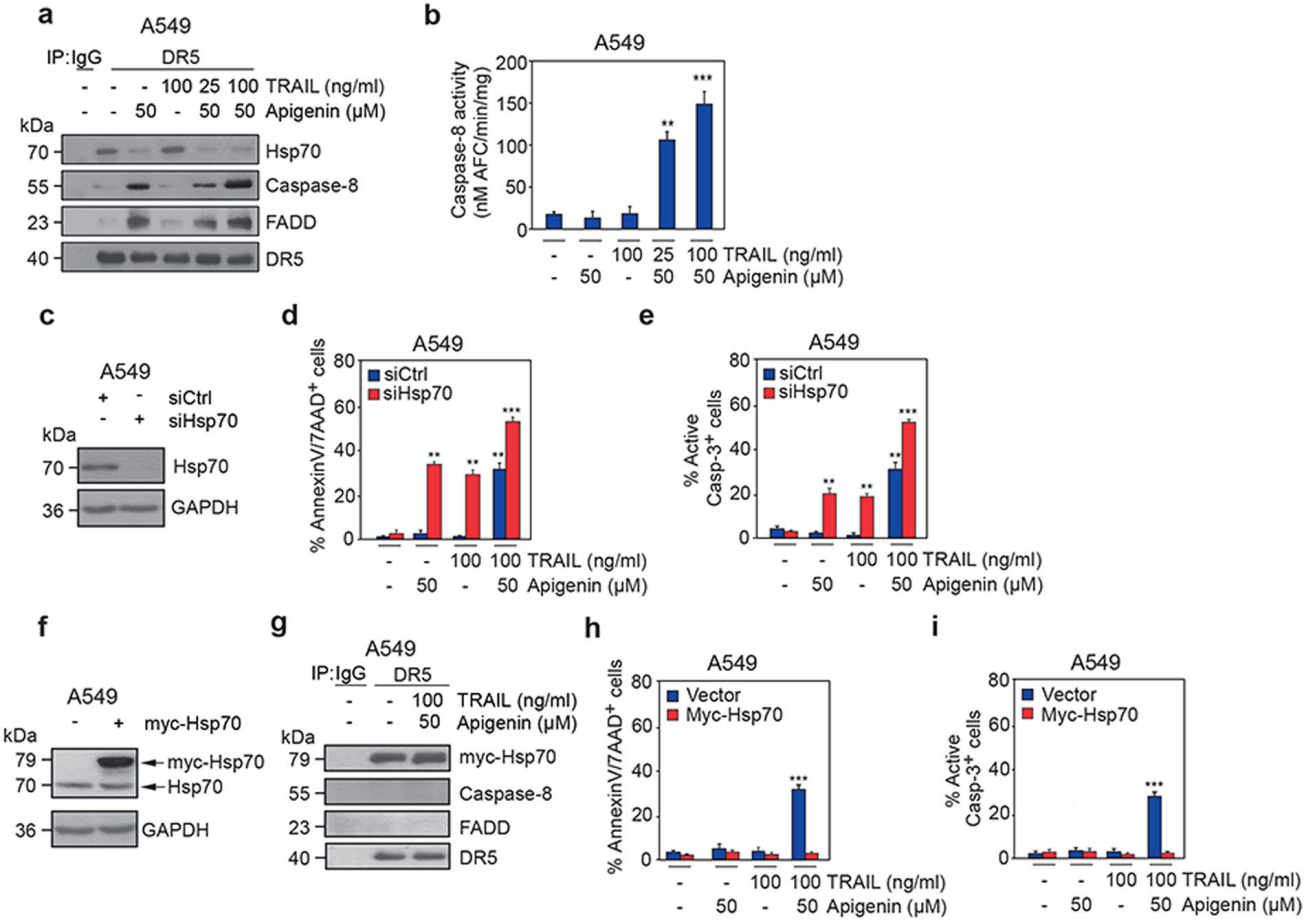
Apigenin binding to Hsp70 increases the responsiveness of lung cancer cells to TRAIL treatment by promoting DISC recruitment to the DR5 receptor. **a** DR5 association with components of the DISC was evaluated by immunoprecipitation using anti-DR5 antibodies (IP: DR5) or an IgG isotype control (IP: IgG) in lysates from A549 cells pretreated with 50 μM apigenin for 18 h followed by 25 or 100 ng/ml TRAIL or DMSO (-) for 6 h or treated with 100 ng/ml TRAIL or DMSO (-) and then immunoblotted with anti-Hsp70, anti-caspase-8, anti-FADD and anti-DR5 antibodies. **b** Caspase-8 activity was determined by IETD-AFC activity assays in the same lysates used in (**a**) and measured as moles of free AFC released per minute reaction time by each mg of protein. **c** Western blot analysis from A549 cells transiently transfected with Hsp70 siRNA (siHsp70) or scramble siRNA control (siCtrl) and immunoblotted with anti-Hsp70 antibodies. The same membrane was reblotted with anti-GAPDH antibodies to measure GAPDH as a loading control. **d** The percentage of apoptotic cells was assessed by flow cytometric analysis of Annexin V/7-AAD in A549 cells that were transiently transfected with siHsp70 or siCtrl and then pretreated with 50 μM apigenin or DMSO (-) for 18 h followed by 100 ng/ml TRAIL or DMSO (-) for 6 h. **e** The percentage of cells with active caspase-3 was evaluated in the cells (**d**) by immunostaining with an antibody that recognized active anti-caspase-3 and determining cell counts by flow cytometry. The data are expressed as the mean ± SEM (*n* = 3; **P < 0.01, and ***P < 0.001; and analyses were performed by one-way ANOVA). **f** Western blot analysis of A549 cells transiently transfected with myc-Hsp70 (+) or pCDNA3-myc vector control (-) and immunoblotted with an anti-Hsp70 antibody, which indicates the levels of overexpressed myc-tagged Hsp70 or endogenous Hsp70. The same membrane was reblotted with an anti-GAPDH antibody to measure GAPDH, which served as a loading control. **g** DR5 association with components of the DISC was evaluated by immunoprecipitation as described in (**a**) using lysates from myc-Hsp70-overexpressing A549 pretreated with 50 μM for 18 h and then with 100 ng/ml TRAIL for 6 h or DMSO (-). Immunoblotting was performed as described in (**a**). **h** The percentage of apoptotic cells was assessed by flow cytometry using AnnexinV/7-AAD in A549 cells transiently transfected with myc-Hsp70 or pCDNA3-myc vector control and pretreated with 50 μM apigenin or DMSO (-) for 18 h and then followed by 100 ng/ml TRAIL or DMSO (-) for 6 h. **i** The percentage of cells with active caspase-3 was evaluated by immunostaining with an antibody that recognizes active caspase-3 and determining cell counts by flow cytometry analysis of eth same cells used in (**h**). All data are presented as the mean ± SEM of three independent experiments (**P < 0.01, and ***P < 0.001; and analyses were performed by one-way ANOVA).

We next investigated how the association of Hsp70 with apigenin in lung cancer cells (Fig. S4) impinges on the assembly of the DISC. We found that in the absence of apigenin, DR5 associated with Hsp70 efficiently, but DR5 binding to FADD and caspase-8 was almost undetectable (Figs. 5a and S6a). Apigenin alone or in combination with TRAIL, almost completely abolished the association of DR5 with Hsp70, while FADD and caspase-8 increased (Figs. 5a and S6a). Consistently, caspase-8 activity was significantly increased with apigenin/TRAIL treatment compared to vehicle, apigenin or TRAIL alone (Figs. 5b and S6b), evidence of increased apoptosis.

We next investigated whether the apigenin-induced assembly of the TRAIL/DISC apoptotic signaling complex was directly mediated by the apigenin-Hsp70 interaction. In A549 cells efficiently transfected with Hsp70 siRNA (siHsp70) (Fig. 5c), apigenin+TRAIL treatment significantly increased the percentage of cells undergoing apoptosis to 60% compared to control siRNA, which was drastically higher than either single treatment (Fig. 5d). In the presence of Hsp70, apigenin/TRAIL treatment-induced apoptosis by ~30%, as evidenced by AnnexinV/7-AAD staining, while neither apigenin nor TRAIL alone affected cell viability (Fig. 5d). Similar results were obtained when active caspase-3 was used as an apoptotic marker (Fig. 5e). Conversely, transient overexpression of myc-Hsp70 in A549 cells (Fig. 5f), was sufficient to inhibit the association of FADD and caspase-8 with DR5 in A549 cells treated with the vehicle and those treated with apigenin and TRAIL (Fig. 5g), and inhibited apigenin+TRAIL-induced apoptosis (Fig. 5h, i). Together, these results demonstrate that apigenin-mediated sensitization to TRAIL-induced apoptosis is regulated by Hsp70.

## Discussion

TRAIL ability to induce apoptosis selectively of cancer cells presents unique opportunities for lung cancer treatment^5,34^. Progress in TRAIL therapy revealed the significance to identify cancer-selective TRAIL-sensitizers able to circumvent the resistance of primary lung cancer cells without negatively affecting normal cells^12^. Here, we identified that splicing reprogramming of TRAIL/DISC components by the dietary flavonoid apigenin is responsible for specific sensitization of primary human lung cancer cells to TRAIL-induced apoptosis. Explicitly, we revealed a mechanism by which apigenin increases the availability of DR5 TRAIL receptor, while weakening the inhibitory effects of c-FLIPs and Hsp70 on the DISC, thereby overcoming the resistance of lung cancer cells to TRAIL-mediated apoptosis.

The use of natural compounds, including flavonoids, as TRAIL-sensitizer agents, is attracting great attention^7^. The specificity of apigenin to induce apoptosis of cancer cells, but having non-toxic effect on nontumor cells or in vivo^18,21^, presents tremendous opportunities for cancer treatment. Here, we found that pretreatment with apigenin sensitizes established human lung cancer cell lines to TRAIL-induced apoptosis (Figs. 1 and S1), in agreement with previous studies administering TRAIL and apigenin concomitantly in lung^25^, leukemia^24^, and prostate cancer cell lines^26^. Notably, we show that apigenin pretreatment specifically sensitizes primary human lung epithelial cells isolated from human adenocarcinomas to TRAIL-induced apoptosis without affecting matched nontumor lung epithelial cells (Fig. 1). These are important findings, as we have shown that pharmacologically-relevant concentrations of apigenin can be successfully delivered by a celery-based food formulated to increase the absorption of apigenin^35,36^, suggesting the possibility of supplementing TRAIL treatment with diets rich in apigenin to improve its clinical performance.

TRAIL resistance is mediated by different mechanisms including reduction of receptors and increase in antiapoptotic regulators, resulting in decreased TRAIL-DISC-dependent apoptosis^37^. Here we demonstrate that apigenin specifically increases the expression of the TRAIL/DISC components DR5 and c-FLIP_S_ without altering FADD or Hsp70 protein levels (Fig. 2), which is likely to contribute to apigenin-induced sensitization to TRAIL-induced apoptosis.

When investigating how apigenin regulates DR5 protein levels we found that total *DR5* steady-state mRNA levels were decreased by apigenin in lung cancer cells, which is consistent with similar findings reported in lymphocytic leukemia^24^. However, these results contrast with recent studies showing that apigenin induces *DR5* mRNA in A549 cells^25^. This discrepancy may reflect preferential amplification of transcript isoforms. Indeed, our results revealed that apigenin decreased *DR5b* transcript levels in tumor cells, while increased *DR5a* transcript, resulting in a *DR5a/DR5b* isoform ratio similar to that commonly observed in nontumor cells (Fig. 3). We showed that the *DR5a* 3′UTR is translated more efficiently than the *DR5b* 3′UTR, which may explain the higher DR5 protein levels in apigenin-treated cells despite the apparently reduced total *DR5* mRNA levels. Consistently, we found higher levels of DR5a protein isoform in apigenin-treated cells (Fig. 3f). Our results provide evidence that apigenin does not affect directly translation (Fig. 3e); rather, it reprograms splicing increasing *DR5a* transcript thereby resulting in higher DR5a protein levels (Fig. 3f) and receptor availability (Supplementary Fig. 3). Similarly, we found that apigenin reprograms *c-FLIP* splicing. *c-FLIPs* was found to be expressed in tumor cells (both primary human PHLEC-ACCs and NSCLC cells) but was absent in nontumor PHLECs (Fig. 4). Previous studies have shown that TRAIL-induced upregulation of *c-FLIPs* was correlated with NSCLC survival^13^. This effect was evident both at the protein and mRNA level suggesting that apigenin-regulation of FLIP isoforms ratio occurred at the splicing level. In addition, we found that the association of apigenin with Hsp70, an inhibitor of the TRAIL/DISC pathway, sequesters Hsp70 from DR5, enabling FADD and caspase-8 recruitment for DISC formation, which triggers caspase-8 activation and results in increased TRAIL-induced apoptosis (Fig. 5). Consistent with the central inhibitory role of Hsp70 in the TRAIL/DISC pathway, silencing of Hsp70 also increased the efficiency of TRAIL monotherapy in inducing cell death. Intriguingly, the lack of Hsp70 also increased apoptosis in cells treated with apigenin alone. These results may be attributable to the ability of Hsp70 to regulate the ability of apigenin to induce apoptosis also through a non-TRAIL-mediated pathway. We showed that apigenin induces apoptosis in cancer cells, including NSCLC, through the intrinsic mitochondria-mediated pathway^21^. Hsp70 prevents BAX translocation to the mitochondria and APAF recruitment^38^, thereby inhibiting caspase-9-dependent activation of the intrinsic apoptotic pathway^39,40^. Together, these results suggest that apigenin sensitization to TRAIL-induced apoptosis is regulated by Hsp70-dependent mechanisms. In addition, recent findings showed that TRAIL/DR axis can promote NFκB-mediated tumor growth and has a tumor-supportive immune-modulatory role^41^. Therefore, it is conceivable that the ability of apigenin and celery-based foods rich in apigenin to inhibit NFκB transcriptional activity and immune-modulate macrophage function^20,35^ may offer additional benefits disabling TRAIL-mediated tumor-supporting immune microenvironment.

**Fig. 6:**
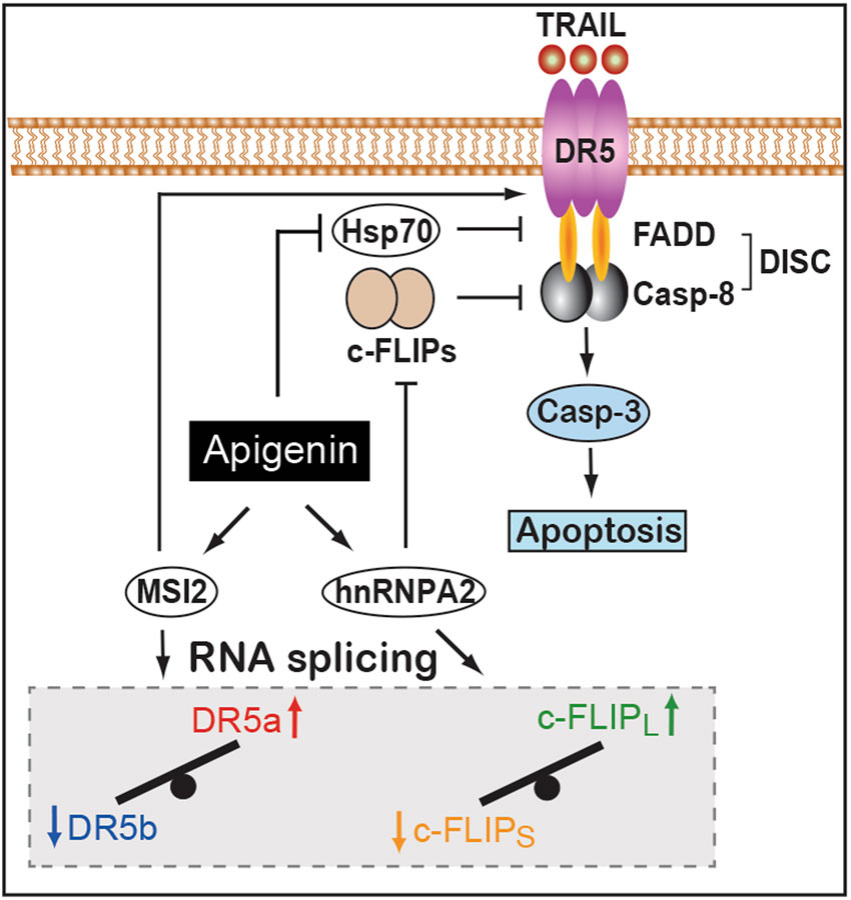
Mechanisms responsible for apigenin-mediated TRAIL sensitization in lung cancer cells. TRAIL signals through the receptor DR5 to activate the DISC (FADD and Casp-8), which culminates in apoptosis. Hsp70 and c-FLIP_S_ inhibit the DISC to block apoptosis. Apigenin reprograms the splicing of DR5 and c-FLIP through MSI2 and hnRPNA2, respectively, resulting in a shift in the predominant isoforms; these shifts impact receptor availability and DISC inhibition, respectively. Further, apigenin directly inhibits Hsp70 as well. Thus, apigenin enables TRAIL-induced apoptosis in lung cancer cells.

We also showed here that apigenin associates with MSI2 and hnRNPA2, which regulate splicing of *DR5* and *c-FLIP*, respectively^33,42^. These observations support a mechanism by which apigenin through these interactions, affects *DR5* and *FLIP* splicing (Fig. 6). The RNA-binding proteins MSI2 and hnRNPA2 are emerging as cancer drivers by affecting the expression of oncogenes^43^ and potentially acting as lung cancer markers^44^. Whether higher levels of MSI2 and hnRNPA2 found in cancer cells might favored their association with apigenin remains to be investigated. Reprogramming of *DR5* splicing by apigenin resulted in higher DR5 protein levels and cell surface availability (Figs. 3 and S3), thereby increasing the ability of TRAIL to trigger the activation of the DISC-mediated apoptotic pathway (Fig. 6). In addition, the inhibition of the DISC complex exerted by c-FLIPs and Hsp70 was released by apigenin through splicing reprogramming and direct association, thereby resulting in increased TRAIL-induced apoptosis (Fig. 6).

Dysregulated alternative splicing affects more than 65% of the cancer proteome and has a considerable impact on treatment resistance^45^. Some flavonoids have been shown to regulate splicing^27,46–50^, while others like genistein showed no effect in splicing^36,51^, suggesting distinct specificities, which due to the fundamental role of diets warrant future investigations.

In conclusion, our findings reveal that dietary apigenin, through a two-pronged mechanism involving splicing reprogramming and direct interaction with Hsp70, leads to increased death receptor availability and cessation of apoptosis inhibition and results in enhanced efficacy of TRAIL-induced apoptosis. These findings that dietary flavonoids can reprogram splicing may have profound implications, which would need further preclinical investigations, for the use of these compounds to overcome resistance and improve cancer treatments.

## Material and methods

### Reagents

Chemicals and flavonoids were from Sigma-Aldrich. Human recombinant TRAIL (Millipore, cat. 616374) was dissolved in TRAIL buffer (500 mM NaCl, 10 mM Na_2_HPO_4_, 2.7 mM KCl, 2 mM KH_2_PO_4_, 0.1 mM DTT, and 10% glycerol).

### Lung cell isolation and culture

Matched lung tumor (T) and adjacent normal tissue (N) biopsies deidentified were obtained from The Ohio State University Comprehensive Cancer Center Biorepository & Biospecimen Resource Department of Pathology with consent and in accordance with the Institutional Review Board guidelines. Lung cells were isolated by GentleMACS Dissociator (Miltenyi Biotec Inc.) using h_tumor_01 followed by 02 programs, followed by filtration and separation onto Ficoll gradient (17-5446-02, GE Healthcare). PHLEC-ACCs or nontumor PHLECs populations were evaluated by immunostaining and flow cytometry using antibodies listed in Supplementary Table 1. Nontumor cells were cultured in DMEM/F12 supplemented with 0.1 ng/ml retinoic acid and PHLEC-ACCs were cultured for 2 weeks in DMEM/F12 supplemented with 30 nM Na-selenite, 10 μM ethanolamine, 10 μM phosphorylethanolamine, 0.5 mM Na-pyruvate, 2 mM glutamine, 0.18 mM adenine, 15 mM HEPES pH 7.2, 5 μg/ml insulin, 5 μg/ml transferrin, 10 ng/ml EGF, 10 ng/ml cholera toxin, 100 nM hydrocortisone, 0.1 nM triiodothyronine, 4 μl/ml bovine pituitary extract, and 5% FBS. All other cells were from American Type Culture Collection.

### Transfections, silencing, apoptosis, viability

Viability assays using MTS (Promega), transient transfections with pCDNA3-myc or pCDNA3-myc-Hsp70 DNA and silencing with 100 nM siHsp70 (SASI_Hs01_00051449, Sigma-Aldrich) or siCtrl (1027284, Qiagen), was done as previously described^52,53^. Apoptosis was evaluated by flow cytometry using AnnexinV-APC and 7-AAD Detection Kit (BD Biosciences) and active caspase-3 immunostaining, as previously described^52^. Caspase-8 activity was determined by evaluating the release of AFC from IETD-AFC, as previously described^54^.

### Protein analyses

Lysates for westerns were obtained with NP-40 buffer (10 mM Tris, pH 7.5, 0.5% NP-40, 1 mM DTT, 0.1 mM PMSF, and 2 μg/ml protease inhibitor cocktail). Immunoprecipitation was done as previously described^52^ using NP-40 buffer with 20 mM Tris-Cl, 150 mM NaCl, 1% NP-40, and 10% glycerol. All antibodies are listed in Supplementary Table 1. Pulldown experiments using A549 cell lysates using apigenin-linked (A) or control (C) beads were conducted as previously described^27^.

### RT-PCR

mRNA was obtained using TRIzol and cDNA using ThermoScript RT-PCR system (Life Technologies). Alternative splicing analyses of splice variants and was done by PCR using isoform-specific primers and resolved by gel electrophoresis. The isoform percentage was calculated by densitometry as follows: 100* (isoform X)/(sum all different isoforms). *DR5* steady-state mRNA levels were detected by RT-PCR. Relative mRNA expression was calculated as 2^-ΔCt(treatment)^/2^-ΔCt(vehicle)^, where ΔCt = (Ct target - Ct internal control). *DR5* mRNA expression was normalized against two internal controls *GAPDH* and actin. Primers are provided in Supplementary Table 2.

### *DR5* 3′UTR luciferase reporter assays

Luciferase activity was evaluated by assessing ONPG (o-nitrophenyl-β-d-galactopyranoside) hydrolysis to o-nitrophenyl formation by spectrometry in transiently transfected pGL3-DR5a 3′UTR, pGL3-DR5b 3′UTR, or pGL3-control-luciferase A549 cells following manufacturers’ recommendations (Promega). All primers used for cloning are listed in (Supplementary Table 2).

### Statistical analyses

Statistical significance is stated within the legends and calculated using GraphPad Prism software version 6.0.

## Supplementary information

Supplementary Figure 1

Supplementary Figure 2

Supplementary Figure 3

Supplementary Figure 4

Supplementary Figure 5

Supplementary Figure 6

Supplementary Figure 7

Supplementary Information
