# Supplementary figures and images for: Splicing reprogramming of TRAIL/DISC-components sensitizes lung cancer cells to TRAIL-mediated apoptosis
Source: Cell Death Dis. 2021 Mar 17;12(4):287. doi: 10.1038/s41419-021-03567-1 (PMC7969956; doi:10.1038/s41419-021-03567-1)

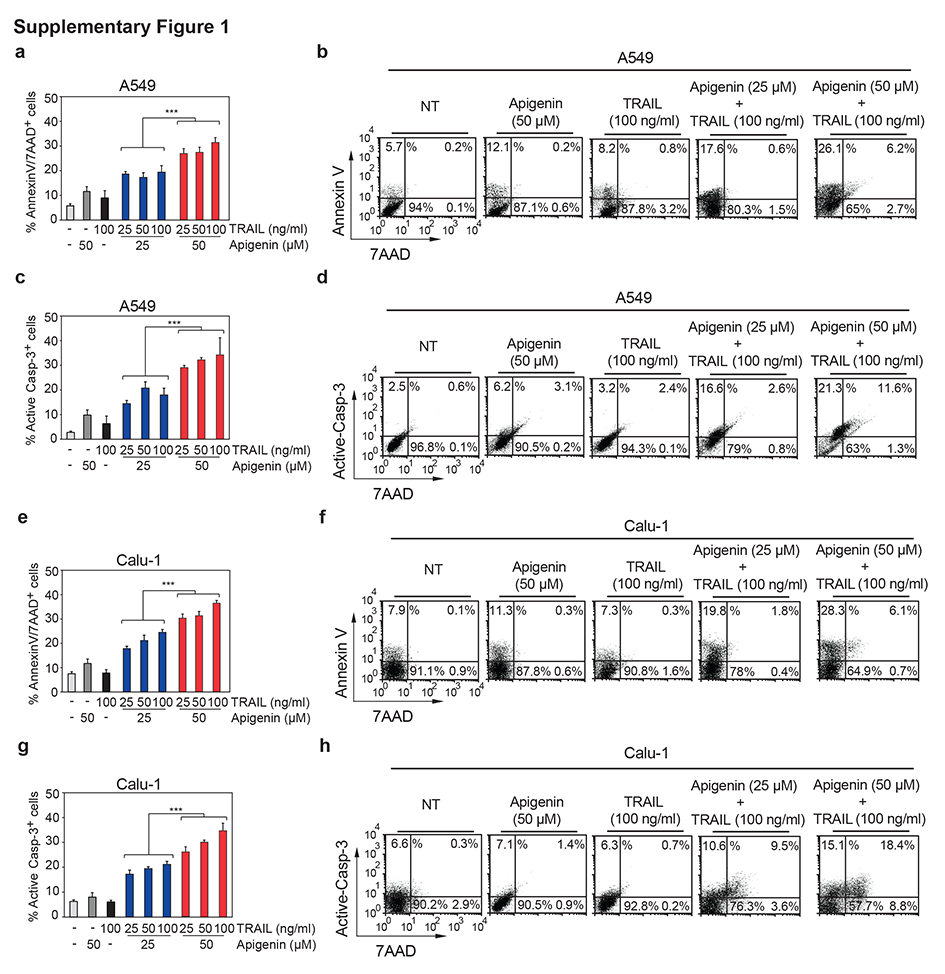

Supplement: Supplementary file 1 — Supplementary Figure 1 [file 41419_2021_3567_MOESM1_ESM.tif]

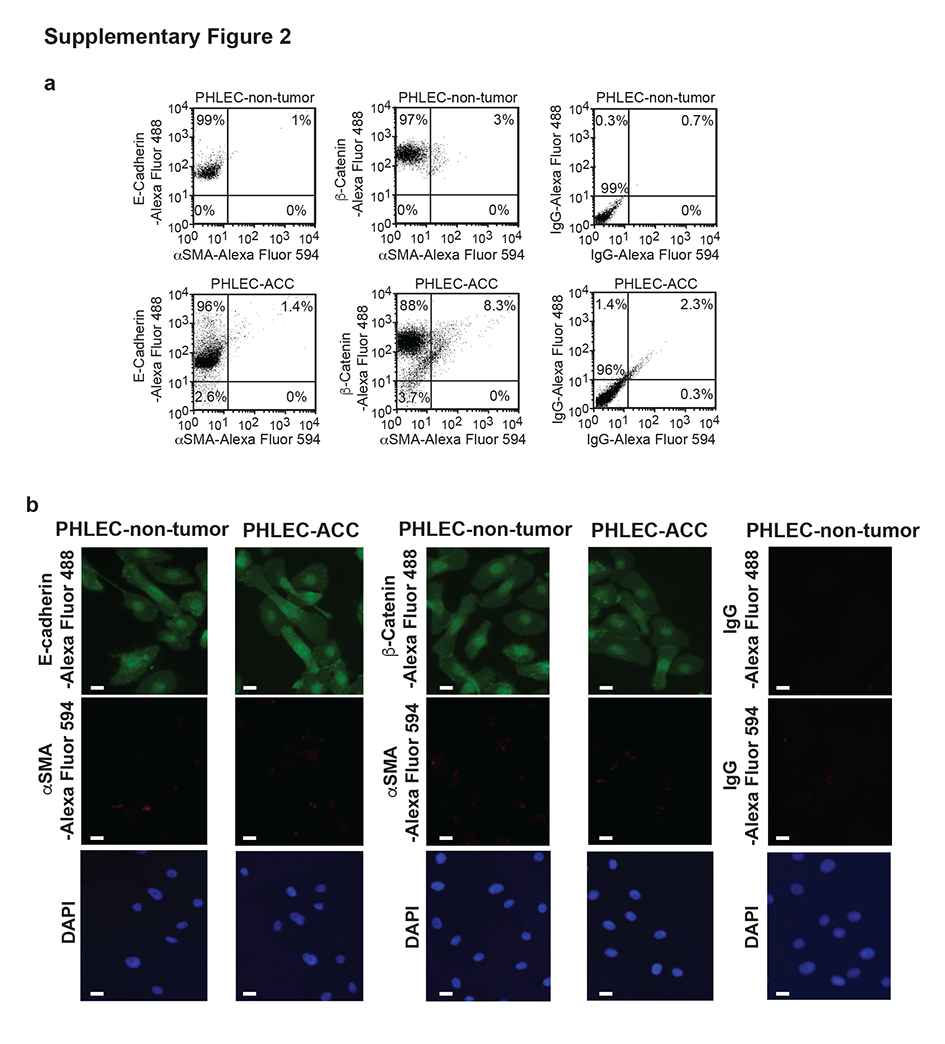

Supplement: Supplementary file 2 — Supplementary Figure 2 [file 41419_2021_3567_MOESM2_ESM.tif]

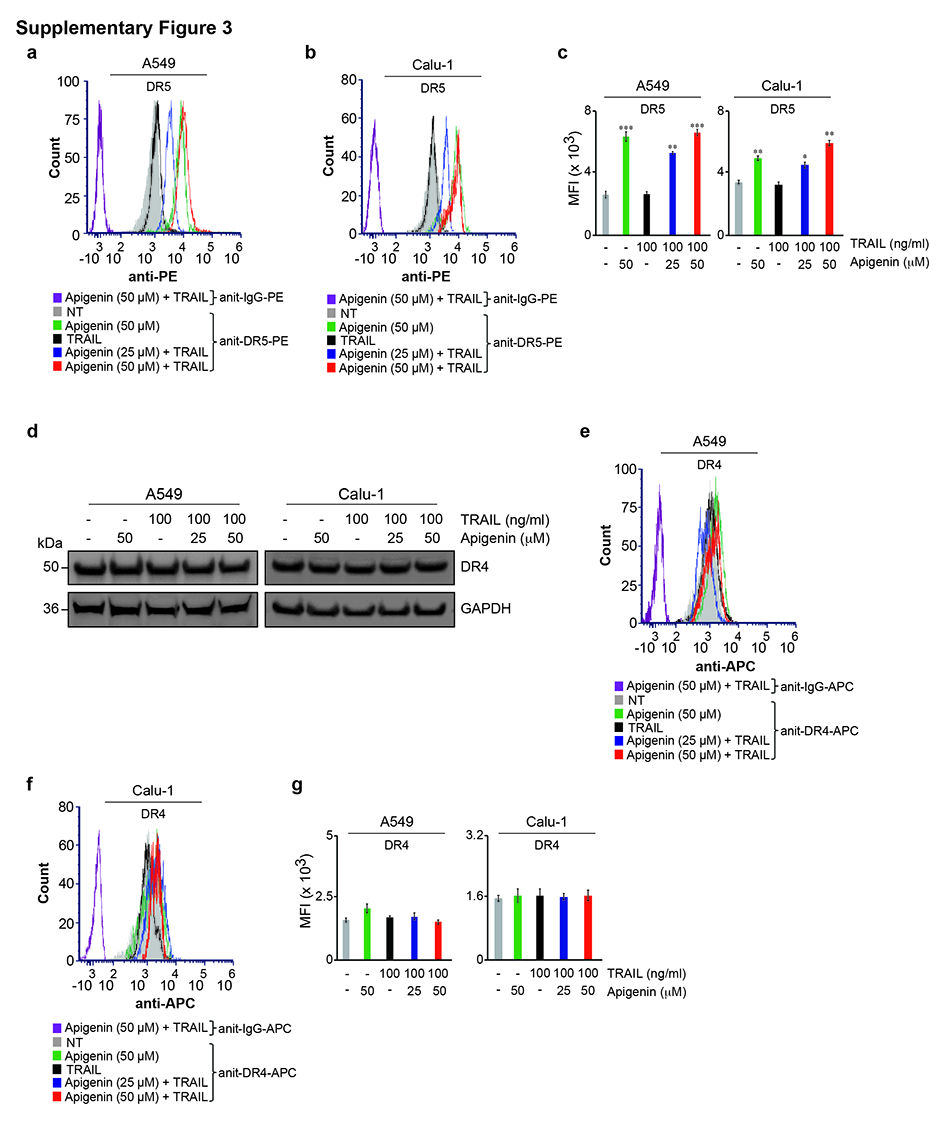

Supplement: Supplementary file 3 — Supplementary Figure 3 [file 41419_2021_3567_MOESM3_ESM.tif]

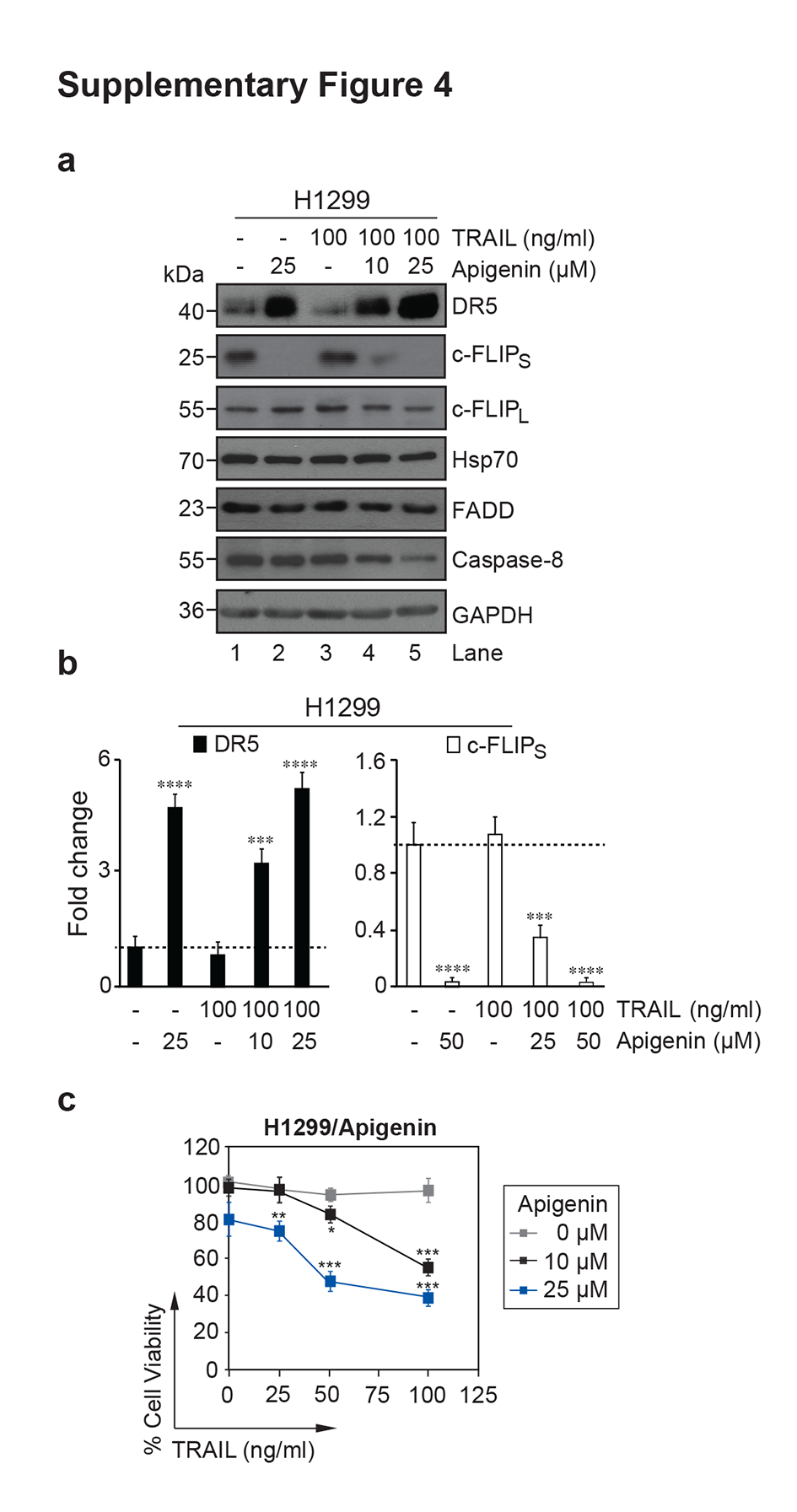

Supplement: Supplementary file 4 — Supplementary Figure 4 [file 41419_2021_3567_MOESM4_ESM.tif]

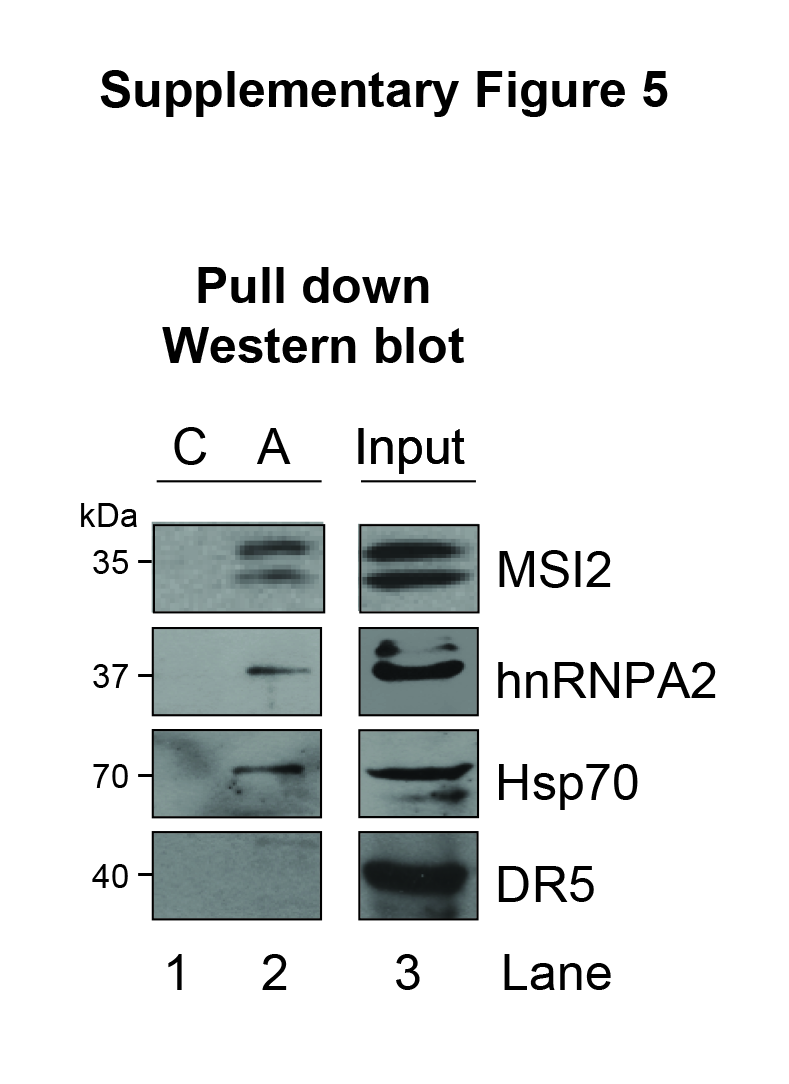

Supplement: Supplementary file 5 — Supplementary Figure 5 [file 41419_2021_3567_MOESM5_ESM.tif]

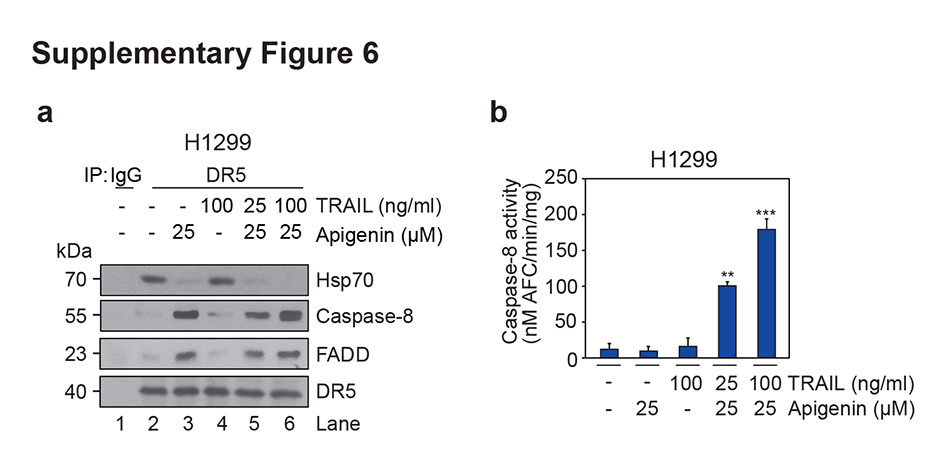

Supplement: Supplementary file 6 — Supplementary Figure 6 [file 41419_2021_3567_MOESM6_ESM.tif]

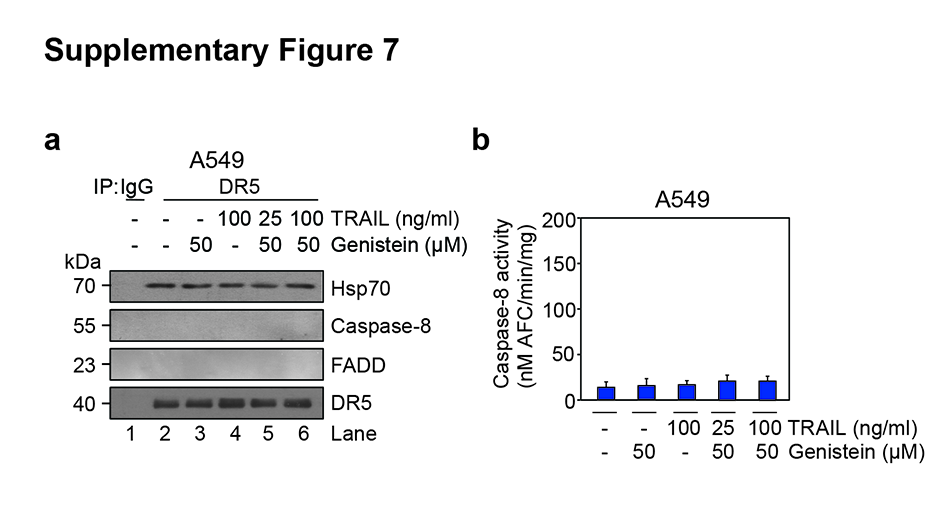

Supplement: Supplementary file 7 — Supplementary Figure 7 [file 41419_2021_3567_MOESM7_ESM.tif]
