# Supplementary material for: Splicing reprogramming of TRAIL/DISC-components sensitizes lung cancer cells to TRAIL-mediated apoptosis
Source: Cell Death Dis. 2021 Mar 17;12(4):287. doi: 10.1038/s41419-021-03567-1 (PMC7969956; doi:10.1038/s41419-021-03567-1)
Supplement: Supplementary file 8 — Supplementary Information [file 41419_2021_3567_MOESM8_ESM.docx]

**Supplementary information**

**Supplementary Figure Legends**

**Supplementary Figure 1. Apigenin increases TRAIL-induced apoptosis of lung cancer cell lines.** Apoptosis was evaluated in A549 (a-d) and Calu-1 (e-g) cells treated with 25 or 50 μM apigenin or DMSO for 18 h and then treated with 100 ng/ml TRAIL for an additional 6 h. **(a)** The percentage of apoptotic A549 cells was assessed by flow cytometry using Annexin V/7-AAD. **(b)** A representative flow cytometry plot of A549 cells used in (a). **(c)** The percentage of A549 cells stained positively with active caspase-3. **(d)** A representative of a flow cytometry plot of a A549 cells used in (c). **(e)** The percentage of apoptotic Calu-1 cells was assessed by flow cytometry using Annexin V/7-AAD. **(f)** A representative flow cytometry plot of Calu-1 cells used in (e). **(g)** The percentage of Calu-1 cells stained positively with active caspase-3. **(h)** A representative flow cytometry plot of Calu-1 cells used in (g). The data are presented as the mean ± SEM (n = 3, ****P* < 0.001, and analyses were performed by two-way ANOVA).

**Supplementary Figure 2. PHLEC isolation from lung tumor human biopsies and matched nontumor tissues. (a)** Representative results of flow cytometry analyses conducted to assess the purity of PHLECs isolated from paired tumor biopsies (PHLEC-ACCs) and normal nontumor tissue biopsies (nontumor PHLECs); cells were stained with anti-β-catenin-Alexa Fluor 488 or anti-E-cadherin-Alexa Fluor 488, both epithelial cells markers, and anti-αSMA-Alexa Fluor 594 antibodies, a fibroblast marker. Anti-IgG-Alexa Fluor 488 and anti-IgG-Alexa Fluor 594 antibodies were used as isotope controls. The percentages of cells shown that are positively labeled by each or both of the tested antibodies are shown. The data indicated that the PHLEC-ACC and nontumor PHLEC populations were >90% pure. **(b)** Representative immunofluorescence images of cells costained with anti-β-catenin-Alexa Fluor 488 or anti-E-cadherin-Alexa Fluor 488 and anti-αSMA-Alexa Fluor 594 antibodies. Nontumor PHLECs stained with anti-IgG-Alexa Fluor 488 and anti-IgG-Alexa Fluor 594 antibodies and used as control of the background signal. DNA was stained using DAPI (blue). Scale bars in (b), 20 μm. The flow cytometry and immunofluorescence results are representative of three independent experiments.

**Supplementary Figure 3. Apigenin increases DR5 cell surface availability, but has no effect on DR4.** DR5 and DR4 cell surface availability were assessed by flow cytometry for A549 and Calu-1 cells. The lung cell lines were pretreated with 25 or 50 μM apigenin for 18 h followed by treatment with 100 ng/ml TRAIL for 6 h, or treated for the whole time with DMSO (NT or -), or 50 μM apigenin alone, or pretreated with DMSO (-) for 18 h followed by TRAIL for 6 h. **(a, b)** Representative results of flow cytometry analyses of A549 (a) and Calu-1 (b) cells stained with PE-conjugated anti-DR5 antibodies or PE-conjugated IgG isotype control. **(c)** Mean fluorescence intensity (MFI) of A549 and Calu-1 cells labeled positive for PE-conjugated anti-DR5 antibodies. **(d)** DR4 protein levels were assessed by western blots in A549 and Calu-1 cells. The same membranes were reblotted with anti-GAPDH antibodies, which functioned as a loading control. The western blot results are representative of three independent experiments. **(e, f)** Representative results of flow cytometry analyses of A549 (e) and Calu-1 (f) cells stained with APC-conjugated anti-DR4 antibodies or APC-conjugated IgG isotype control. **(g)** Mean fluorescence intensity (MFI) of A549 and Calu-1 cells labeled positive for APC-conjugated anti-DR4 antibodies shows no changes in the expression of DR4. All data (c and g) are presented as the mean ± SEM (n = 3; **P* < 0.05, ***P* < 0.01, and ****P* < 0.001; and analyses were performed by two-way ANOVA).

**Supplementary Figure 4. Apigenin modulates the expression of DISC components. (a, b)** Protein levels of DISC components in H1299 human NSCLC cells treated with 10 or 25 μM apigenin for 18 h followed by 100 ng/ml TRAIL for 6 h. The effects of 25 μM apigenin and 100 ng/ml TRAIL and DMSO (-), as well as the aforementioned cotreatments, were assessed by immunoblotting with specific anti-DR5, anti-c-FLIP_S_, anti-c-FLIP_L_, anti-Hsp70, anti-FADD, and anti-caspase-8 antibodies. The same membranes were reblotted with anti-GAPDH antibodies to establish a loading control. The western blot results are representative of three independent experiments. **(b)** Densitometry was performed using ImageJ, and the results are presented as the fold change ratio of the protein of interest over GAPDH. The value for the control treatment (-) was established as 1 to enable easy comparison of relative values. (c) MTT assays were used to assess the viability of H1299 cells treated with DMSO (vehicle) or 10 or 25 μM apigenin for 18 h and then 25, 50 or 100 ng/ml TRAIL for an additional 6 h. The data are presented as the mean ± SEM (n = 3; **P* < 0.05, ***P* < 0.01, ****P* < 0.001, and *****P* < 0.005; and analyses were performed by two-way ANOVA).

**Supplementary Figure 5. Apigenin associates with the RNA-binding proteins MSI2, hnRNPA2 and Hsp70.** Lysates of A549 cells were incubated with either apigenin-linked (A) or control (C) beads, and proteins that were pulled down were identified by anti-MSI2, anti-hnRNPA2, anti-Hsp70 and anti-DR5 antibodies, as analyzed by western blotting. The input lane indicates total lysates that was not subjected to a pulldown experiment. The data are representative of three independent experiments.

**Supplementary Figure 6.** **Apigenin enhances TRAIL sensitivity in NSCLC H1299** **cells by promoting DISC member association with the DR5 receptor via dissociation of Hsp70. (a)** Association of DISC members with DR5 was assessed by immunoprecipitation (IP) experiments, which were performed using H1299 cell lysates and anti-DR5 or anti-IgG isotype control antibodies. First, cells were treated with 25 μM apigenin or DMSO (-) for 18 h, which was followed by treatment with 25 or 100 ng/ml TRAIL for 6 h; other cells were treated with either 25 μM apigenin or 100 ng/ml TRAIL or DMSO (-). The samples were resolved by SDS-PAGE and immunoblotted with anti-caspase-8, anti-Hsp70, anti-FADD and anti-DR5 antibodies. **(b)** Caspase-8 activity was determined by AFC activity assay using protein lysates from cell treatments shown in (a). The data are presented as the mean ± SEM (n = 3; ***P* < 0.01, and ****P* < 0.001; and analyses were performed by one-way ANOVA).

**Supplementary Figure 7. Genistein affects neither recruitment of DISC components to the DR5 receptor nor TRAIL-induced apoptosis. (a)** DR5 association with components of the DISC was evaluated by immunoprecipitation of DR5 (IP: DR5) using an anti-DR5 antibody or an IgG isotype control (IP: IgG). Immunoprecipitations were performed using lysates from A549 cells treated with 50 μM genistein for 18 h and then 25 or 100 ng/ml TRAIL or DMSO (-) for 6 h or treated with 50 μM genistein, DMSO (-) or 100 ng/ml TRAIL. Then, analyses of immunoprecipitations were performed by immunoblotting with anti-Hsp70, anti-caspase-8, anti-FADD and anti-DR5 antibodies. **(b)** Caspase-8 activity was determined by IETD-AFC activity assays using the same lysates as in (a), and measured as moles of free AFC released per minute reaction time by each mg of protein. The data are presented as the mean ± SEM (n = 3; no significant difference; and analyses were performed by one-way ANOVA).

**Supplementary Material and methods**

**Table 1: Antibodies and sources of antibodies used in this study**

| Antibodies name | Clone | Company | Used for |
| --- | --- | --- | --- |
| anti-GAPDH | FL-355 | Santa Cruz, Dallas, TX | Western blot analyses |
| anti-Myc | 9E10 | Santa Cruz | Western blot analyses |
| anti-hnRNPA2/B1 | DP3B3 | Santa Cruz | Western blot analyses |
| anti-FADD | 2782 | Cell Signaling, Danvers, MA | Western blot analyses |
| anti-MSI2 | EP1305Y | Abcam, Cambridge, MA | Western blot analyses |
| anti-c-FLIP | 7F10 | Enzo Life Sciences, Farmingdale, NY | Western blot analyses |
| anti-Hsp70 | C92F3A-5 | Enzo Life Sciences | Western blot analyses |
| anti-full-length caspase-8 | MAB4708 | Millipore, Burlington, MA | Western blot analyses |
| anti-DR5 (detects 40 kDa DR5a and 48 kDa DR5b protein isoforms) | 2019 | ProScience, Poway, CA | Western blot analyses |
| anti-DR4 | 24063-1-AP | Thermo Fisher Scientific, Waltham, MA | Western blot analyses |
| HRP-conjugated anti-mouse |  | GE Healthcare, Piscataway, NJ | Western blot analyses |
| HRP-conjugated anti-rabbit |  | GE Healthcare | Western blot analyses |
| anti-DR5 (detects DR5a isoform) | AB16942 | Millipore | Western blot analyses/ Immunoprecipitation |
| anti-mouse IgG |  | Santa Cruz | Immunoprecipitation |
| anti-rabbit IgG |  | Santa Cruz | Immunoprecipitation |
| anti-αSMA-Alexa Fluor594 | 1A4 | Abcam | Immunofluorescence assay |
| anti-β-Catenin-Alexa Fluor488 | E247 | Abcam | Immunofluorescence assay |
| anti-E-Cadherin-Alexa Fluor488 | HECD-1 | Abcam | Immunofluorescence assay |
| anti-IgG-Alexa Fluor488 |  | Abcam | Immunofluorescence assay |
| anti-IgG-Alexa Fluor594 |  | Abcam | Immunofluorescence assay |
| PE-conjugated anti-human CD262 (DR5) | DJR2-4(7-8) | BioLegend, San Diego, CA | Flow Cytometry analyses |
| APC-conjugated anti-human CD261 (DR4) | DJR1 | BioLegend | Flow Cytometry analyses |
| PE-conjugated anti-IgG |  | BioLegend | Flow Cytometry analyses |
| APC-conjugated anti-IgG |  | BioLegend | Flow Cytometry analyses |
| Anti-active FITC-conjugated caspase-3 |  | BD Biosciences, San Jose, CA | Flow Cytometry analyses |
| anti-CD16/32 | 2.4G2 | BD Biosciences | Flow Cytometry analyses |

**Table 2: List of primers used in this study**

| Primer name | Sequence (5’- 3’)  Forward (F) / Reverse (R) | Used for |
| --- | --- | --- |
| PAO-230 | F: ACTTTGGTATCGTGGAAGGACT | *GAPDH detection* |
| PAO-231 | R: GTAGAGGCAGGGATGATGTTCT | *GAPDH detection* |
| PAO-403 | F: GCGGGAAATCGTGCGTGACATT | *β-actin detection* |
| PAO-404 | R: GATGGAGTTGAAGGTAGTTTCGTG | *β-actin detection* |
| PAO-545 | F: CTTGGCCAATTTGCCTGTAT | *c-FLIP_L_ detection* |
| PAO-546 | R: GGCAGAAACTCTGCTGTTCC | *c-FLIP_L_ detection* |
| PAO-547 | F: CGAGGCAAGATAAGCAAGGA | *c-FLIP_S_ detection* |
| PAO-548 | R: CACATGGAACAATTTCCAAGAA | *c-FLIP_S_ detection* |
| PAO-802 | F: AAGACCCTTGTGCTCGTTGT | *DR5 detection* |
| PAO-803 | R: AGGTGGACACAATCCCTCTG | *DR5 detection* |
| PAO-916 | R: CACCTTCTAGAGTGTGATTCTCTTCAGGAAGTCAGACCTTCCCT | *DR5a* and *DR5b* 3’UTRs amplification from A549 |
| PAO-917 | F: TCTAGAAACGACTGTGTAGATGGATCTTACAATGTAGCCCA | *DR5a* 3’UTR amplification from A549 |
| PAO-918 | F: TCTAGAATTTTTATATAAGGTTTCATATTTAATTTGGTCATGGA | *DR5b* 3’UTR amplification from A549 |
| PAO-799 | F: AGAGGGATTGTGTCCACCTG | *DR5a* and *DR5b* splice variants |
| PAO-1024 | R: TACGGCTGCAACTGTGACTC | *DR5a* and *DR5b* splice variants |
| PAO-1003 | F: TTTTGTATTTTGTGTAGATATAGGGGCTCT | Multi site-directed mutagenesis |
| PAO-1004 | R: CTAGAAGATGCATGTAGAGTGTCAGCCTTAT | Multi site-directed mutagenesis |
